# *Myrica esculenta* Leaf Extract—Assisted Green Synthesis of Porous Magnetic Chitosan Composites for Fast Removal of Cd (II) from Water: Kinetics and Thermodynamics of Adsorption

**DOI:** 10.3390/polym15214339

**Published:** 2023-11-06

**Authors:** Anjali Yadav, Sapna Raghav, Nirmala Kumari Jangid, Anamika Srivastava, Sapana Jadoun, Manish Srivastava, Jaya Dwivedi

**Affiliations:** 1 Department of Chemistry, Banasthali Vidyapith, Banasthali 304022, India; raoanjli001@gmail.com (A.Y.);; 2 Department of Chemistry, Nirankari Baba Gurubachan Singh Memorial College, Sohna 122103, India; 3 Departamento de Química, Facultad de Ciencias, Universidad de Tarapacá, Avda. General, Velásquez, Arica 1775, Chile; sjadoun@academicos.uta.cl; 4 Department of Chemistry, University of Allahabad, Prayagraj 211002, India

**Keywords:** adsorption, cadmium removal, chitosan, MNPs, wastewater treatment

## Abstract

Heavy metal contamination in water resources is a major issue worldwide. Metals released into the environment endanger human health, owing to their persistence and absorption into the food chain. Cadmium is a highly toxic heavy metal, which causes severe health hazards in human beings as well as in animals. To overcome the issue, current research focused on cadmium ion removal from the polluted water by using porous magnetic chitosan composite produced from Kaphal (*Myrica esculenta*) leaves. The synthesized composite was characterized by BET, XRD, FT-IR, FE-SEM with EDX, and VSM to understand the structural, textural, surface functional, morphological-compositional, and magnetic properties, respectively, that contributed to the adsorption of Cd. The maximum Cd adsorption capacities observed for the Fe_3_O_4_ nanoparticles (MNPs) and porous magnetic chitosan (MCS) composite were 290 mg/g and 426 mg/g, respectively. Both the adsorption processes followed second-order kinetics. Batch adsorption studies were carried out to understand the optimum conditions for the fast adsorption process. Both the adsorbents could be regenerated for up to seven cycles without appreciable loss in adsorption capacity. The porous magnetic chitosan composite showed improved adsorption compared to MNPs. The mechanism for cadmium ion adsorption by MNPs and MCS has been postulated. Magnetic-modified chitosan-based composites that exhibit high adsorption efficiency, regeneration, and easy separation from a solution have broad development prospects in various industrial sewage and wastewater treatment fields.

## 1. Introduction

Water is the main component for the survival of all living organisms. However, it is gravely endangered due to the massive amount of pollution caused by domestic, industrial, and agricultural actions. Water scarcity and quality have emerged as major issues for long-term development [1,2]. Many water contaminants, including organic and inorganic constituents, have been mentioned in the literature [3]. Heavy metal-contaminated water bodies pose serious problems owing to their toxic behavior and bioaccumulation [4,5,6,7].

Cadmium is one of the non-essential heavy metals that is regarded as highly noxious and carcinogenic due to its non-biodegradability and ability to bioaccumulate in the environment. It is classified as a human carcinogen by the Environmental Protection Agency, (EPA), US, with a maximum permissible limit of 0.005 mg/L in drinking water [8].

Various methods for removing Cd (II) from the aqueous environment that is often used include chemical precipitation, membrane treatment, ion exchange, electro-dialysis, membrane flotation, electrochemical methods, ultra-filtration, etc. [9]. However, these processes, have several limitations, including lower effectiveness, high energy requirements, expensive disposal, and incomplete removal. Adsorption is extensively used for wastewater treatment due to its, quick response, low cost, simple operation, absence of sludge production, and reusability [10,11,12].

Various types of adsorbents have been used to remove Cd from wastewater. Among them, natural polysaccharide polymers, particularly chitosan, and its derivatives, have gained the attention of researchers owing to their renewable nature, sustainability, and adsorption efficiency [13,14,15]. Chitosan (CS), which is formed by the deacetylation of chitin, is present in the shells of crustacean crabs and shrimps, insect carapaces, and fungal and plant cell walls. CS, as one of nature’s most abundant biopolymers, has sparked widespread scientific interest due to its low cost, non-toxicity, inadequate hydrophilicity, biocompatibility, and biodegradability. It has many functional groups, including -NH_2_ and -OH, which facilitate good sorption of heavy metal ions. Raw chitosan, like many other widely used materials, cannot be efficiently separated from aqueous medium using traditional separation methods. As a result, attempts have been made to recycle CS by combining it with magnetic nanoparticles [16,17]. Because magnetic chitosan composites benefit from both chitosan (excellent adsorption performance) and magnetic material (easy magnetic separation), combining CS with a magnetic component is an effective way to address the above-mentioned inadequacies [18,19].

**Figure 1:**
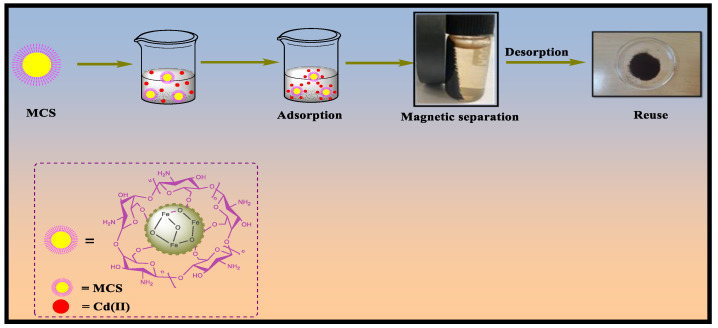
Systematic illustration of magnetic chitosan composite for Cd(II) removal.

Due to their high chelating capacity and ease of magnetic separation, porous magnetic chitosan (MCS) material has been considered an efficient adsorbent for Cd removal [20]. A schematic representation of the application of MCS for the adsorption of Cd (II) and the reuse of the adsorbent is provided in Figure 1. The combination of MNPs and CS successfully excludes chitosan’s inherent drawbacks for application as an adsorbent, and its separation and reuse. Furthermore, the -OH groups on the surface of MNPs can form a hydrogen bonding network with chitosan by interacting with its -OH and -NH_2_ groups. As a result, the stability and integrity of the composite are retained even under acidic or alkaline environments [21]. Various chitosan-based materials were synthesized with magnetic NPs because of their lower internal diffusion resistance and higher specific surface area. MNPs, on the other side, get easily oxidized in air and are chemically active resulting in aggregation and loss of magnetism. Because of their small size, these NPs cause secondary contamination. Magnetic cores with larger sizes, on the other hand, can aid in the magnetic separation process [22]. Porous Magnetic chitosan composites have been used extensively and successfully to eliminate pollutants such as dyes, heavy metal ions, and other organic contaminants.

During the modification process, functional groups such as hydroxyl and carboxyl groups increased. Various studies are performed for the removal of Cd (II) by magnetically modified material, and the results showed that after the material was magnetically modified, the pH value, specific surface area, and polar oxygen-containing functional groups all increased, resulting in a saturated adsorption capacity. The mechanism of MCS to remove Cd is surface complexation and electrostatic adsorption. The strong affinity of iron oxide for Cd can enhance the complexation between them.

Plant extracts have been proposed as an easy and convenient alternative to chemical and physical methods for the preparation of metallic nanoparticles and their composites in recent years owing to the concerns of sustainability and environmental deterioration using conventional non-renewable sources. MNPs are one of these nanoparticles that have caught the interest of many researchers. *Myrica esculenta* leaf extract contains a variety of bioactive phytoconstituents, including phenolic compounds, glycosides, alkaloids, triterpenoids, and volatile oils [23].

To the best of our knowledge, there has not been much research done to assess the effectiveness of magnetic chitosan employing leaf extract of *Myrica esculenta* (Kaphal) for removing cadmium from aqueous solutions. Therefore, our aim is to develop a green synthetic process to produce chitosan—Fe_3_O_4_ composite using *Myrica esculenta* leaf extract as a reducing agent. This has resulted in the development of a novel porous magnetic chitosan composite in the present work that turned out to be an efficient material for Cd (II) ion removal. The MCS composite as well as the Fe_3_O_4_ nanoparticles were characterized using XRD, BET sorptometry, FT-IR, FE-SEM, EDX, and VSM for evaluating their physicochemical properties and also for determining the effect of such properties on the adsorption process parameters, namely, contact time, pH, adsorbent dose, and initial concentration of the analyte (Cd^2+^). The potential of MNPs and magnetic chitosan composite as potential adsorbents for Cd (II) was further probed using kinetic models and adsorption isotherms. Moreover, the reusability of the composite was investigated up to seven cycles.

## 2. Materials and Method

### 2.1. Materials

FeCl_2_·4H_2_O, Chitosan (degree of deacetylation ≥ 95%; viscosity: 100–200 mPa.s), FeCl_3_·6H_2_O, and acetic acid (CH_3_COOH), used during the preparation for adsorbents were of analytical-grade and were procured from Sigma Aldrich (St. Louis, MO, USA). Cd (NO_3_)_2_·4H_2_O was purchased from E. Merck, Mumbai, India. All aqueous solutions for the adsorption studies were prepared using Millipore Milli-Q^®^ ultrapure water (Jaipur, Rajasthan). As Cd (II) has been chosen as the model contaminant in this investigation, a solution of Cd (II) of desired concentrations was prepared and used as an adsorbate solution. Dried Kaphal leaves were sourced from the farmlands of Uttarakhand.

### 2.2. Preparation of Aqueous Leaf Extract of Myrica esculenta (Kaphal)

**Figure 2:**
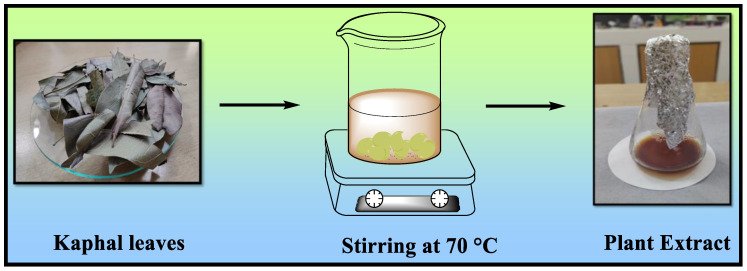
Preparation of plant extract of *Myrica esculenta* (Kaphal).

To remove any pollutants, the Kaphal leaves were thoroughly washed with distilled water after being collected from the highlands of Uttarakhand, India. The leaves were manually cut into little pieces, air-dried for a week at room temperature, and then manually ground in a home kitchen grinder; 4 g of the resulting fine powder was then added into double-distilled water and stirred at 70 °C for 45 min. It was then filtered, and the filtrate was stored at 4 °C (Figure 2). The synthesis process for Fe_3_O_4_ nanoparticles and Fe_3_O_4_/Chitosan Composite is shown in the Supplementary file.

### 2.3. Characterization of the Adsorbents

The XRD of the MNPs and the CS composite were recorded on (Bruker D8 Discover X-ray Diffractometer, Karlsruhe, Germany) using Cu Kα radiation. The XRD pattern was recorded in the 2θ range of 10° and 70°.

By using a surface area analyzer N_2_ adsorption desorption studies were carried out on Quantachrome Autosorb iQ Surface Analyzer, CIQTEK, Hefei, China). For the analysis of the surface area, the samples were first degassed for 3.0 h to remove volatile gases and were then placed in a surface area analyzer for N_2_ adsorption-desorption.

A Perkin-Elmer FT–IR spectrometer was used to record the FTIR spectra. A spectrum of the adsorbents in the range of 400–4000 cm^−1^ to characterize the nature of chemical bonding and the type of surface functional group.

FE-SEM was used to investigate the morphology of MNPs and MCS composites as well as their elemental composition. FE-SEM equipped with EDAX (FEI QUANTA FEG250, Oregon, OH, USA) and an INCA Energy X-MAX-50, Oregon, OH, USA) was employed to characterize the morphology and chemical composition of the adsorbents used in the study.

A LakeShore 7404 (Lakeshore cryotronics, Westerville, OH, USA) vibrating sample magnetometer was used to determine the magnetization properties of the adsorbents. The magnetic sample is positioned on the sample holder and placed between the electromagnetic poles, normally horizontally, for VSM measurement.

### 2.4. Batch Adsorption Studies for the Adsorption of Cd (II) Using MNPs and Chitosan/Fe_3_O_4_ Composite as Adsorbents

To study the optimal adsorption performance and investigate the adsorption mechanism, batch adsorption experiments were performed. Different parameters were examined in fixed ranges, including, adsorbent dose (0.01–0.1 g), pH (2–11), temperature (303, 313, and 323 K), contact time (5–50 min), initial metal concentration (10–100 mgL^−1^), and adsorption/desorption studies, to better understand the potential of MNPs and MCS as adsorbents for Cd (II). The C_0_ (initial concentration) values were used to conduct the adsorption isotherms. After the adsorption process, atomic absorption spectroscopy was utilized to examine the heavy metal ion concentration remaining in the filtrate. The removal effectiveness (%) and equilibrium adsorption capacity (q_e_) of the adsorbents, namely, MNPs and MCS composite. For Cd (II) was given by Equations (1) and (2).

(1) $$\%\;Adsorption=\frac{\left(C_{0}-C_{e}\right)\times 100}{C_{0}}$$

(2) $$Adsorption\;Capacity\left(q_{e}\right)=\frac{\left(C_{0}-C_{e}\right)\times V}{m}$$

where, V is the volume of metal ion solution, m is the mass of adsorbent dose, C_0_ is the initial metal ion concentrations, C_e_ is the residual Cd (II) ion concentration, and q_e_ is the adsorption capacity at C_e_, respectively.

## 3. Result and Discussion

### 3.1. Adsorbent Characterization

#### 3.1.1. X-ray Diffraction

**Figure 3:**
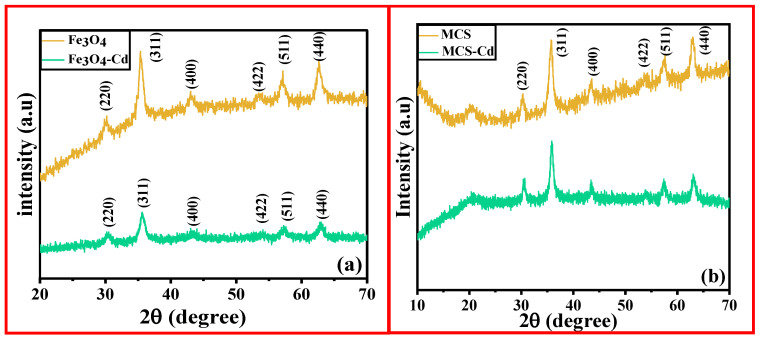
XRD spectra for (**a**) MNPs [before and after Cd (II) adsorption], (**b**) Porous MCS adsorbent [before and after Cd (II) adsorption].

To understand the chemical and physical structure of the magnetic particles encapsulated in the CS matrix, X-ray diffraction (XRD) is a highly valuable technique. Figure 3a,b represent the XRD patterns of MNPs and porous MCS composite. The crystalline phase of magnetic NPs and porous magnetic chitosan composite were analyzed by XRD.

The diffraction peaks at 2θ values of 30.2°, 35.6°, 43.2°, 53.6°, 57.2°, and 62.9° correspond to the (220), (311), (400), (422), (511), and (440) planes of MNPs (JCPDS file number 01-075-0033) [24]. These peaks closely resemble the standard XRD pattern of Fe_3_O_4_. Thus, the XRD pattern demonstrated the formation of magnetic particles. Diffraction peaks at 2θ values in the range of 21° to 28° were attributed to amorphous chitosan (Figure 3b). Moreover, the peaks typical of Fe_3_O_4_ were also present in the composite as expected [25].

#### 3.1.2. BET Sorptometry for Evaluating the Textural Properties of Adsorbents

**Figure 4:**
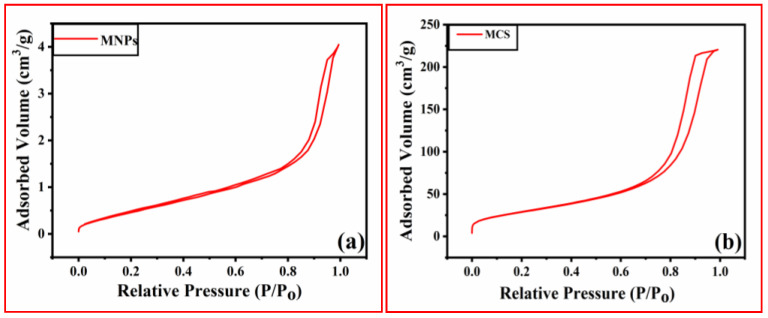
N_2_ sorption isotherm of (**a**) MNPs, (**b**) MCS composite.

N_2_ gas adsorption-desorption isotherms were observed in the relative pressures (p/p_0_) values ranging from 0 to 1 to estimate the surface area and porosity values of the synthesized adsorbents. BET sorptometry was performed to investigate the average pore radius, surface area, and pore volume of the material. About 25.0 mg of material was degassed at 300 °C for analysis. According to IUPAC standards, the N_2_ adsorption-desorption isotherms are Type IV, which agrees with the mesoporous nature of the composite [26]. The surface area and total pore volume of MNPs and MCS were 105 m^2^/g, 173 m^2^/g, and 0.3410 cc/g, and 0.4305 cc/g, respectively (Table 1). The Langmuir surface area of the MNPs and MCS was 201.084 m^2^/g and 536.934 m^2^/g, respectively (Figure 4). Thus, the higher surface area is responsible for the higher adsorption capacity of porous MCS compared to MNPs.

The Langmuir curve and BET multipoint of MNPs and porous MCS are expressed in detail in the Supporting Information (Figure S3).

#### 3.1.3. Fourier Transform Infrared (FT-IR) Spectroscopy

**Figure 5:**
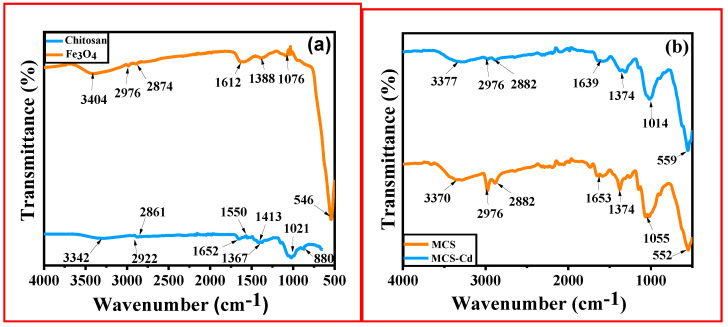
TIR spectra for (**a**) Chitosan and Fe_3_O_4_ nanoparticles, (**b**) MCS adsorbent [before and after Cd (II) adsorption].

For understanding the nature of chemical bonding and the kind of surface functionalities the adsorbents used in this study were examined using FT-IR spectroscopy. The FT-IR spectra of chitosan and MNPs are shown in Figure 5a. Likewise, the FT-IR spectra of the MCS composite before and after the adsorption process are shown in Figure 5b.

The absorption bands in the range of 3200–3400 cm^−1^ corresponded to N–H and O–H stretching vibrations of CS (Figure 5a). The bands at 2922 cm^−1^ and 2861 cm^−1^ were due to the C-H symmetric and unsymmetric stretching vibrations of the –CH_2_ groups in CS. The -NH deformation, C-N axial deformation, -CH_3_ bending vibration, and stretching vibration of C-O-C in the chitosan structure appeared at, around 1413, 1367, and 1021 cm^−1^. The existence of chitosan in the composite and its structural integrity was confirmed by the appearance of peaks at 1652 cm^−1^ and 1550 cm^−1^, which corresponds to the N-H bending vibration of primary amine [27].

The absorption bands characteristic of MNPs were observed in the FT-IR spectrum (Figure 5a). Metal oxygen bonds typical of MNPs were observed in the range of 400 and 850 cm^−1^. The absorption band at 546 cm^−1^ was attributed to the Fe-O stretching vibration of MNPs. This band is very sharp and has strong intensity, indicating the crystallinity of the sample. The broad bands around 3404 cm^−1^ and 1612 cm^−1^ were due to the O–H stretching vibration of surface-adsorbed water molecules. The band located around 1388 cm^−1^ and 1076 cm^−1^ were attributed to the unsymmetric and symmetric stretching vibrations of COO- [28].

Similar peaks were observed in the FT-IR spectrum of the MCS composite. In addition, some specific bands correspond to the original formation of new bonds between chitosan and Fe_3_O_4_ and some bands of chitosan have moved slightly from their original positions owing to atomic-level interactions between chitosan and MNPs. In the FT-IR spectrum of the MCS composite (Figure 5b), specific bands were observed at 3370 cm^−1^ which was attributed to N-H stretching vibration. The band at 1652 and 1550 cm^−1^ was attributed to C=O stretching vibration in the CS, for the N-H scissoring from the primary amine due to free amino groups in the CS and was compared with the standard chitosan [29]. The peak at 552 cm^−1^ for the Fe-O group was due to bare magnetic nanoparticles. This spectrum (Figure 5b) unambiguously showed the presence of both CS and MNPs in the composite [30]. The NMR spectrum of *Myrica esculenta* leaf extract has been already discussed by Nguyan et al. [31].

#### 3.1.4. FE-SEM-EDX

**Figure 6:**
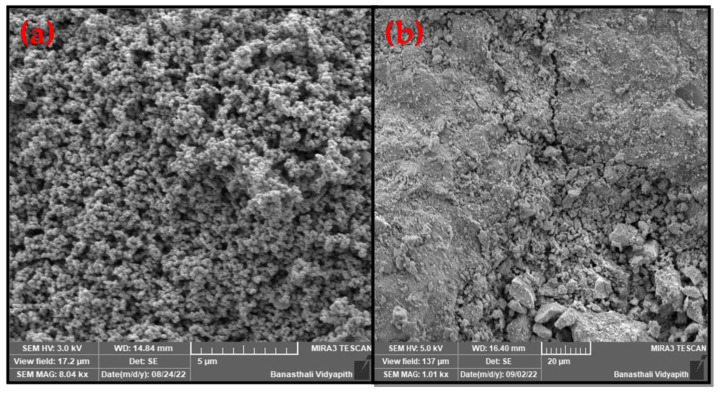
FE-SEM image of (**a**) MNPs, and (**b**) MCS composite.

FE-SEM was used to find out the morphology of MNPs and MCS composite, and EDAX was utilized to determine the elemental composition. SEM and EDX images of both the adsorbents were displayed in Figure 6 (EDX result shown in Figure S4). Spherical-shaped particles with agglomeration were observed in synthesized magnetic NPs (Figure 6a) as well as MCS (Figure 6b) composite in the FE-SEM images. Fe_3_O_4_ NPs exhibited a smooth surface, and the particles had an irregular shape, as observed in Figure 6a. In Figure 6b, the FE-SEM image of MCS was shown to exhibit a rough, granular surface after chitosan was bonded to Fe_3_O_4_. Agglomeration of the particles on the surface of the composite cannot be ruled out [32]. EDX spectra of the Fe_3_O_4_ sample confirm the presence of iron (Figure S4). The EDX spectra of MNPs and MCS are shown in Supporting Information (Figure S4). The mass percentage of carbon in MCS composite is higher than in MNPs, which proved MCS composite had lesser hydrophilicity properties.

#### 3.1.5. Vibrating Sample Magnetometer Study for Evaluating the Magnetic Property of the Adsorbents

**Figure 7:**
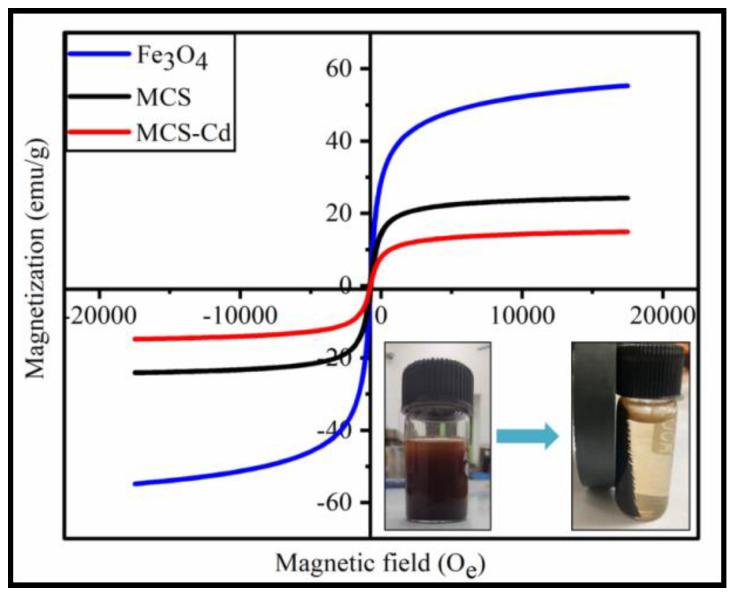
Magnetization curves of the Fe_3_O_4_, MCS composite, and MCS composite after Cd (II) adsorption.

To evaluate the adsorbents’ magnetic properties, VSM (Vibrating-sample magnetometer) tests were utilized [20]. The magnetic properties of investigated magnetic materials were studied by measuring the magnetization curves (Figure 7). The results showed that all magnetization curves pass through the origin, which indicated that there was no residual magnetization occurring in test samples and these materials have super-paramagnetism [33]. By changing H between O_e_ +10,000 and −10,000 O_e_, magnetization hysteresis was produced. MNPs had a saturation magnetization of 55.070 emu/g. The saturation magnetization of MCS decreased to 24.186 emu/g after the formation of composite between MNPs and chitosan, indicating that the adsorbent was still super-paramagnetic and that it would be easier to separate the adsorbent from solution by gravity and magnetism within a short period of time [34]. The lower M_s_ value for MCS could be explained by the quenching of surface magnetic moment in the material owing to the presence of non-magnetic species, such as CS as expected [35]. Even though MCS’ saturation magnetization value was obviously lower than that of MNPs, the application of a magnet allowed the adsorbent to immediately aggregate and to be separated in the 20 s [32].

### 3.2. Adsorption Experiments

#### 3.2.1. Study of Kinetics of Adsorption of Cd (II) onto the Adsorbents, MNPs, and MCS Composite

To find out the appropriate rate expression and adsorption mechanism, the adsorption kinetics was examined [36]. Several models were examined for the adsorption of contaminants in water onto the surface of adsorbents and also to identify the main mechanism of such adsorption process and the kinetics of adsorption [37]. Adsorption kinetics was investigated in this work by fitting Lagergren’s pseudo-first-order (PFO) and Ho’s second-order models. The adsorption process was determined using the Elovich and intra-particle diffusion (IPD) models [38].

The PFO kinetic model represents weak interaction between sorbate and sorbent predominantly proceeding via physisorption. The PFO kinetic model is represented below [39]

ln (q_e_ − q_t_) = lnq_e_ − k_1_ × t (3)

PSO kinetic model is based on chemisorption. Pseudo–second order kinetics is represented as [40].

t/q_t_ = 1/(k_2_ × q_e_^2^) + t/q_e_ (4)

The IPD model is given by Equation (5).

q_t_ = k_id_ × √t + C (5)

The Elovich model is specified by Equation(6).

q_t_ = (1/β) ln (α × β × t) + (1/β) lnt (6)

where, k_1_ (min^−1^) and k_2_ (g/mg/min) are the rate constants of PFO and PSO, k_id_—intraparticle diffusion rate constant (mg/(gmin^−0.5^)), constant C is the y-intercept, α is the initial rate of adsorption, β is the desorption constant [41].

Four kinetic models were studied, and the kinetic parameters deduced from various models are summarized in Table S1 [42,43].

**Figure 8:**
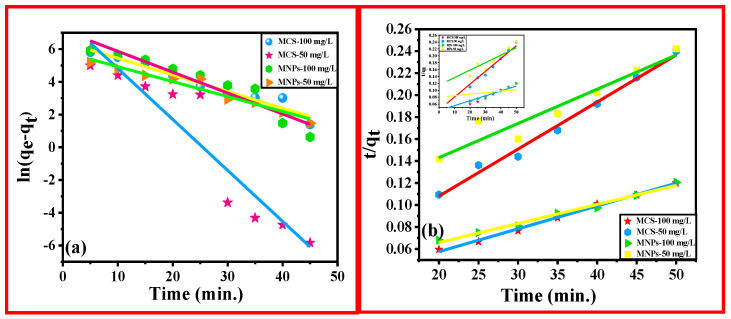
(**a**) PFO and (**b**) PSO for MNPs and MCS composite.

From the R^2^ analysis of kinetic models, the best-fitted model for Cd (II) adsorption on MNPs at 50 mg/L was Elovich, and at 100 mg/L was IPD. Also, from the R^2^ analysis of kinetic models, the PSO model was the best fit for MCS. The pseudo-second-order model’s correlation coefficient (R^2^) was observed to be significantly greater than other models employed for MCS adsorbent, which means that the mechanism of adsorption was governed by this model (Figure 8).

#### 3.2.2. Adsorption Isotherm Models

Adsorption isotherms describe how adsorbents and adsorbates interact in aqueous media at the attained saturation point. The most popular isotherm models, including Langmuir, Freundlich, Dubinin–Radushkevich, and Temkin isotherm models, were utilized.

The Langmuir isotherm is characterized by single-layer sorption onto a surface with countless open sites without interaction between the adsorbate molecules under the assumption that the material is regular and homogeneous. The model’s primary problem is its assumption that the energies of the adsorbent sites at each location are uniform. Equation (7) describes the Langmuir isotherm’s linear for [44]

(7) $$\frac{1}{q_{e}}=\frac{1}{K_{L}q_{max}C_{e}}+\frac{1}{q_{max}}$$

where q_max_ represents the material’s maximum adsorption capacity, and K_L_ is the Langmuir adsorption constant (L/mg) representing the attraction of binding sites [45].

R_L_, the dimensionless constant separation factor is employed to express the important characteristic of the Langmuir isotherm:

$$R_{L}=\frac{1}{1+{K_{L}C}_{0}}$$

**Figure 9:**
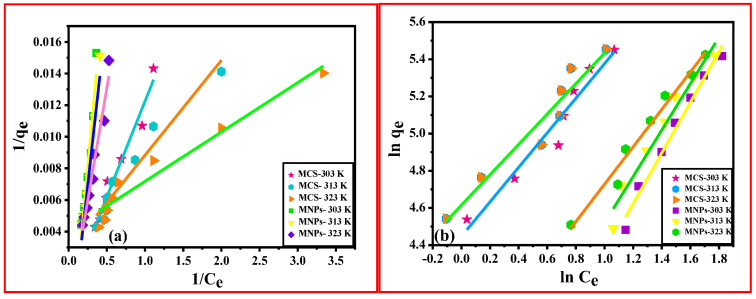
(**a**) Langmuir, (**b**) Freundlich curves for Fe_3_O_4_ and MCS composite as adsorbents for Cd (II) at different temperatures.

The value of R_L_ shows whether the adsorption isotherm is favorable, linear, or unfavorable. The value of R_L_ was found in the range of 0.0717–0.898 for the MCS composite. This shows the efficient interaction between the MCS composite and cadmium ions [46]. Figure 9a shows the Langmuir plot for MNPs and MCS composite. The values of K_L_ and q_max_ are calculated using the slope and intercept of the linear regression plot of 1/C_e_ vs. 1/q_e_, which also provides the R^2^ value, which indicates how well the experimental results correspond with the mathematical isotherm model.

If adsorption happens on heterogeneous surfaces, the Freundlich adsorption isotherm is an empirical equation that is utilized to describe multilayer (physisorption) adsorption as well as monolayer (chemisorption) [42].The linearized Freundlich isotherm equation is explained by Equation (8).

(8) $$Logq_{e}=\log K_{F}\;+\;\frac{1}{n}\log C_{e}$$

where K_F_ is the Freundlich constant of the adsorbent, and n is the adsorption intensity of the adsorbent. 1/n is the adsorption intensity which signifies the heterogeneity of the adsorbent sites as well as the relative distribution of energy. K_F_ and n are dimensionless constants. The value of n > 1 and 1/n < 1 indicates the favorable condition for adsorption. In this study, the value of n ranged between 1.075 and 1.222 which is greater than 1 for MCS composite, indicating favorable adsorption. The graph of lnC_e_Vs lnq_e_ gives a straight line with intercept K_F_ and slope 1/n as shown in Figure 9b. The R^2^ values are 0.97, 0.95, and 0.97 for MNPs at 303, 313, and 323 K and 0.94, 0.92, 0.92 for MCS at 303 K, 313 K and 323 K (Table S2) [45].

The Temkin Isotherm Model was used to characterize the adsorption considering the interaction between adsorbate and adsorbent, which resulted in a linear reduction in isotherm when the heat of adsorption of all molecules in the layer was included Equation (9) provides the isotherm:

q_e_ = βlnK_T_ + βlnC_e_ (9)

where, β = RT/b.

β represents the heat of adsorption (J/mol) and K_T_ is the Temkin isotherm constant (L/g). The plot of q_e_Vs lnC_e_ produces a straight line with slope β and intercept βlnK_T_. *b* is the Temkin constant which is associated with the sorption heat (J/ mg) (Figure S6).

The adsorption is characterized by a uniform distribution of binding energies. The binding energies were 199.28, 185.86, and 146.10 J/mol for MNPs and 132.85, 94.68, and 69.43 J/mol for MCS at 308, 303, and 298 K, respectively [46,47,48].

The D-R isotherm can be used to find out the adsorption mechanism.

Dubinin-Radushkevich (D-R) isotherm (Dubinin, 1960) is:

lnq_e_ = lnq_m_ − βε^2^ (10)

ε^2^ and b are constants. The D-R constants q_m_ and b were calculated from the slope and intercept of lnq_e_Vs ε^2^ [37]. The q_m_ calculated by the D-R model was 640.699 mg/g and 598.7594 mg/g for MNPs and MCS composite at 303 K, respectively (Figure S4).

Four isotherm models were studied, and the significant parameters are shown in Table S2.

### 3.3. Thermodynamic Studies

To investigate the spontaneity, feasibility, and endo/exothermic nature, thermodynamic data plays an important role. The effect of solution temperature (20–50 °C) on Cd (II) ion adsorption was investigated under optimal conditions. Standard entropy (S), enthalpy (H), and Gibbs free energy (G) were calculated as thermodynamic parameters. The Van’t Hoff equation was used to calculate these thermodynamic parameters.

(11) $$K_{d}=\frac{q_{e}(w/v)}{C_{e}}$$

lnK_d_ = ΔS^0^/R − ΔH^0^/RT (12)

ΔG^0^ = −RT lnK_d_ (13)

where ΔG^0^ is the Gibbs free energy, ΔS^0^ is the entropy, and ∆H^0^ is the enthalpy, K_d_ is the distribution coefficient for the adsorption process.

**Figure 10:**
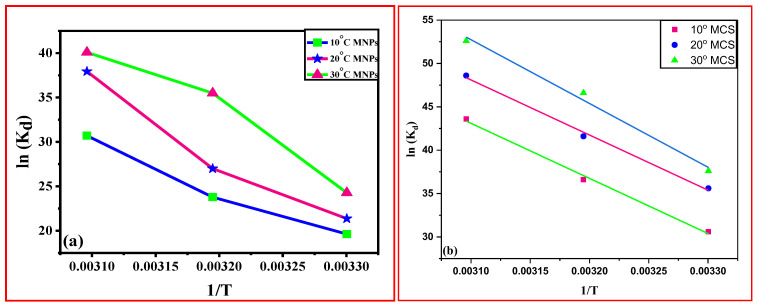
Linear dependence of ln (Kd) on 1/T based on adsorption thermodynamics for (**a**) MNPs and (**b**) MCS at various temperatures.

Equation (11) can be used to calculate the value of K_d_ after the values of q_e_ and C_e_ have been determined experimentally. Using the value of K_d_ in Equation (12), the values of enthalpy and entropy can be calculated. Using Equation (12), the slope and intercept of the plot ln (K_d_) vs. 1/T in Figure 10 would give the values ∆H^0^ and ∆S^0^, respectively.

From Table S3, the positive value of ΔH shows that the adsorption process is endothermic, which confirms the chemisorption nature of adsorption. The negative value of ΔG at all three temperatures for MNPs and at 303 K for MCS composite indicated that the spontaneity of the process was favored at these temperatures. The positive value of ΔS showed a rise in randomness during the adsorption of Cd (II).

### 3.4. Reusability of Adsorbent

**Figure 11:**
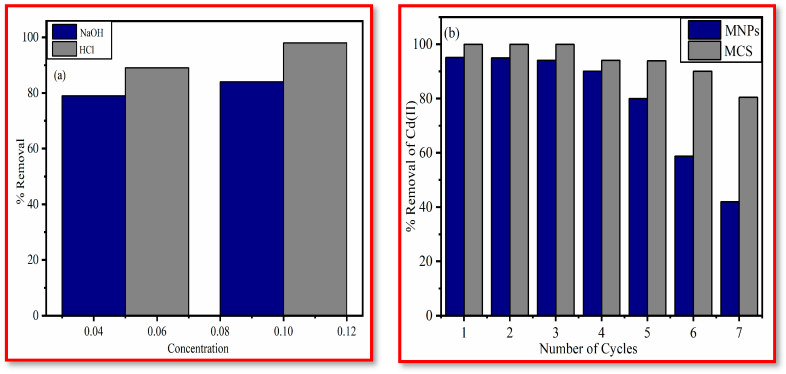
Regeneration studies using (**a**) 0.1 M HCl and 0.1 M NaOH, (**b**) Regeneration studies of MNPs and MCS (0.1 M HCl).

Metal ion desorption from the sorbent and adsorbent regeneration are critical challenges in terms of adsorbent reusability. The major goals of the regeneration process are to recover useful components from the adsorbed phase and to restore the sorption capability of the exhausted material [49]. Figure 11 displays the results of seven adsorption-regeneration cycles (Figure 11a,b). Using various agents including HCl (0.05 M, 0.1 M), and NaOH (0.05 M, 0.1 M), the batch technique was used to evaluate the desorption of the sorbed cadmium from Fe_3_O_4_ and MCS composite. It was discovered that 0.1 M HCl produced the greatest amount of Cd (II) desorption (98%) while using 0.1 M NaOH, the desorption efficiency was observed to be 84%. Adsorption, as well as desorption cycles, were repeated seven times utilizing the same adsorbents to assess the adsorbent’s reusability. After each cycle of adsorption, the solid portion of the adsorbent was centrifuged and further mixed in DI water. This mixture was then agitated for about 1/2 h. The remaining suspension was once again used for a different batch experiment. Seven rounds of a similar process were performed. The removal capacity of the regenerated sorbent gradually reduced in comparison to the original adsorbent. As a result, MCS was an effective reusable adsorbent that could be used to recover Cd (II) ions from aqueous medium. The percentage removal of Cd (II) decreased from the first to seventh cycle (i) 99.99% to 80.44% for MCS, (ii) 95.1% to 42% for MNPs. This demonstrates that the MCS may be reused at least seven times while retaining good sorption efficiency. As a result, it was possible to conclude that magnetic chitosan composite can be employed as an efficient material in the removal process.

### 3.5. Comparative Studies

MnFe_2_O_4_/CS microspheres were prepared by coating chitosan on MnFe_2_O_4_. Maximum sorption capacity was observed to be 60.6 mg/g for Cd (II) removal. The experiments showed that the composite could maintain sorption capacities after three cycles of adsorption–regeneration [50]. A new nanobiosorbent based on methionine-glutaraldehyde Schiff’s base-modified cross-linked chitosan magnetic beads was prepared by Salehi et al. (2020) for the elimination of Cd (II). The utmost sorption capacity was observed to be 163.9 mg/g [51]. Chitosan and sodium tripolyphosphate cross-linked chitosan beads were synthesized for cadmium removal from an aqueous medium by Babakhani and Sartaj (2020). The maximum sorption capacity was observed to be 99.87 mg/g [52]. Attapulgite/CoFe_2_O_4_@SiO_2_-chitosan/EDTA was synthesized by solvothermal and sol-gel techniques. The maximum sorption capacity was observed to be 127.79 mg/g [53]. Fan et al. (2017) studied the sorption of cadmium by using magnetic chitosan nanoparticles. The maximum sorption capacity was observed to be 36.42 mg/g for Cd (II). Relatively, the maximum sorption capacity for pure Fe_3_O_4_ nanoparticles was observed to be 13.04 mg/g [54]. Li et al. (2017) prepared chitosan/polyethylenimine grafted magnetic gelatin to eliminate cadmium from wastewater. According to the Cd (II) sorption results, the process can be explained by a monolayer forming on the surface of the material with 321 mg/g from the Langmuir isotherm [55]. The relative adsorption capacity of various adsorbents for Cd (II) and the relative adsorption performance of MCS-based adsorbents for heavy metal ions removal are shown in Table 2 and Table 3, respectively.

### 3.6. Adsorption Studies

#### 3.6.1. Variation of Adsorbent Dose

**Figure 12:**
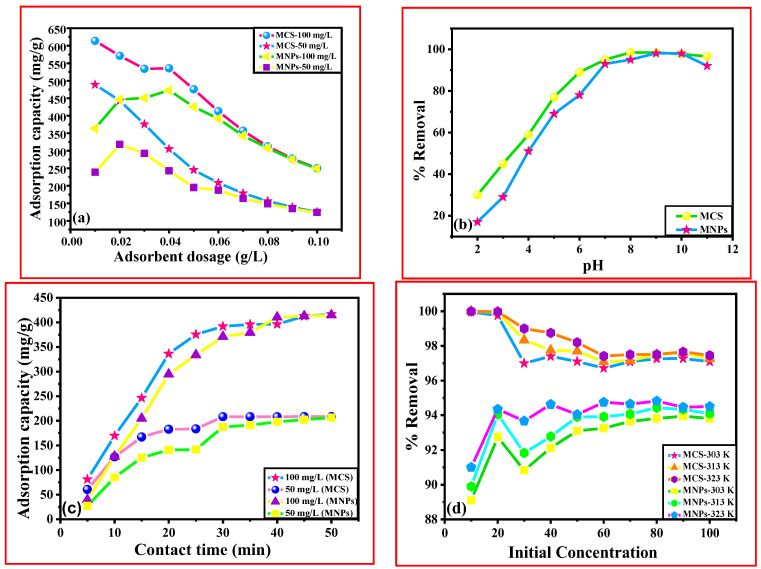
Effect of (**a**) adsorbent dose, (**b**) pH, (**c**) contact time, (**d**) initial concentration for adsorption of Cd (II) on Fe_3_O_4_ and MCS composite, respectively.

The amount of the adsorbent used is a crucial factor in calculating sorption capacity. Studies were carried out by changing the sorbent dosage from 0.01 to 0.1 g/L, while all other factors such as contact duration, temperature, and rpm were held constant to optimize the sorbent dose for the elimination of Cd (II) ions from the aqueous medium. Adsorption capacity experienced a substantial reduction with increasing adsorbent doses. This may be explained by the fact that a greater sorbent dose makes it easier for Cd (II) ions to access active sites on the pores of magnetic chitosan composite, which enhances removal rates. More easily accessible functional groups and adsorption sites for metal ions were responsible for the increment. However, higher adsorbent dosage most certainly enhances particle interactions, like aggregation, which is due to high sorbent concentration. As a result, the adsorbent’s active surface area is significantly reduced, which lowers its capacity for adsorption. The adsorption process achieves the equilibrium point due to the overcrowding of adsorbent particles brought on by the overlapping of adsorption sites after a gradual decline in the sorption capacity of Cd (II) ions from the wastewater. The decline in the ratio of Cd (II) per mass unit of sorbent might potentially account for this [20,22]. The adsorption capacity for MCS fell from 613.75 mg/g to about 249.99 mg/g for C_o_(100) and from 488.7 mg/g to 124.99 mg/g for C_o_(50) as the sorbent dosage rose from 0.01 g/L to 0.1 g/L. Also, the adsorption capacity for MNPs fell from 363.75 mg/g to 248.75 mg/g for C_o_(100) and from 238.75 mg/g to 123.875 mg/g for C_o_(50) as the sorbent dosage rose from 0.01 g/L to 0.1 g/L, as shown in Figure 12a.

#### 3.6.2. Variation in pH

The solution’s pH is a very important factor that strongly affects the sorption process because it influences the charge of the surface by protonation and deprotonation of the material and degree of ionization. By conducting equilibrium adsorption experiments at various pH levels, the impact of the suspending medium’s pH on Cd (II) removal was investigated. In the current study, the influence of solution pH on the sorption of Cd (II) by MNPs and MCS was examined throughout a pH range of 2–11 (Figure 12b). Temperature, dose, rpm, dosage, and other factors were all held constant. Low adsorption efficiency was found at low pH which was caused by competition between the Cd (II) ion and the H^+^ ion for placements on material active sites. Additionally, the concentration of adsorbent positive charges is large in acidic conditions, which results in electrostatic repulsion between the magnetic composite and the Cd (II), decreasing the efficiency of Cd (II) elimination. The removal of cadmium reduces when the pH rises from 2 to 6 because the positive charges on the adsorbent surface are reduced, repulsive forces are stronger, and the positive charges are less attractive [74]. The electrostatic attraction was strengthened as the pH increased, which progressively raised the degree of deprotonation of functional groups and improved the sorption capacity. The utmost removal was seen between pH 6.0 and 8.0 [75].

#### 3.6.3. Variation of Contact Time

It has a pronounced effect on the elimination of adsorbate species from aqueous medium. To assess the Cd (II) adsorption behavior by MNPs and magnetic chitosan composite, the influence of time of contact between the adsorbent and adsorbate was optimized in this work by altering the contact duration from 5 to 50 min. By separating the supernatant at different times, the effect of contact time on sorption capacity was measured. The adsorption rate was quick in the beginning, as seen in Figure 12c. The initial strong absorption of Cd (II) ion was owing to high adsorbate and adsorbent interactions with low solute-solute interaction and more adsorption site accessibility. As the time increased, equilibrium was attained in the adsorption as more and more active sites were occupied. However, after equilibrium was reached, mass transfer diminished and the repulsion of the adsorbate molecule on the surface and inside the solution increased.

It took around 50 min to achieve equilibrium for uptake of Cd (II) [76]. The increment in time can offer plenty of chances for Cd(II) to adsorb to the composite surface. Figure 12c shows that on raising the contact time from 5 to 50 min, the sorption capacity increased from 81.458 to 416.65, 60.625 to 208.33, 39.79 to 414.79, and 27.29 to 206.458 mg/g for MCS (100 mg/L), MCS (50 mg/L), MNPs (100 mg/L), and MNPs (50 mg/L).

The results demonstrated that the adsorbents showed fast removal of Cd (II) within 50 min which is faster when we compare with the reported literature of other adsorbents employed for the Cd (II) removal [77].

#### 3.6.4. Variation of Concentration

The elimination of Cd (II) by adsorption on MNPs and magnetic composite (MSC) was examined in relation to the effect of initial concentration. It appears that the metal ion’s initial concentration is significant and influences the sorption capacity. Because the initial concentration of Cd (II) may offer the driving force required to transfer a mass of Cd (II) between the water phase and the adsorbent surface, the concentration of Cd (II) can also impact the performance of the sorption process.

With the rise in the concentration of Cd (II), there was a decrease in % removal. The experiments were performed at different initial concentrations varying from 10 to 100 mg/L. The graph (Figure 12d) illustrates that with an increase in the concentration of Cd (II) ions, there is a decrease in Cd (II) removal % and a rise in sorption capacity. The presence of active surfaces and sites in the MNPs and MCS composite structure for the assignment of Cd (II) can be related to improving efficiency at low initial concentrations of Cd (II). Additionally, when the concentration of cadmium rises, the %-age of Cd (II) removal decreases, which is explained by the saturation of the MSC’s active sites above a specific level of Cd (II), at which point the system enters equilibrium and no further Cd (II) adsorption takes place [22].

The % removal of Cd (II) by adsorption onto the adsorbent decreases from 99.96 to 97.09, 99.99 to 97.25, and 99.999 to 97.45 for MCS (303 K, 313 K, and 323 K) and from 89.1 to 93.81, 89.9 to 94.1, and 91.0 to 94.5 for MNPs (303 K, 313 K, and 323 K), respectively (Figure 12d).

## 4. Adsorption Mechanism

The Cd (II) ion has an empty d orbit, and both nitrogen and oxygen atoms have lone electron pairs that can attach metal ions to create the complex via electron pair sharing. Fe_3_O_4_ enclosed in CS NPs and MNPs not only provides rich functional groups (-OH, -COOH, and -CO-NH-) as a binding site for heavy metal ions but also has a greater specific surface area to maximize functional group utilization. Functional groups on Fe_3_O_4_-loaded CS NPs and MNPs are freely available for metal ion coordination bonding.

Since negatively charged adsorbents might be quickly absorbed by positively charged groups, such as –OH^2+^ and –NH^+^_3_ in acidic conditions, the electrostatic attraction was a potential adsorption mechanism. The negatively charged functional groups like COO- and AOA on the surface of Fe_3_O_4_ -loaded CS NPs and the Cd (II) ion may establish an electrostatic interaction. Therefore, under the influence of both coordination bonds with the oxygen atom in Fe_3_O_4_ and electrostatic attraction, the adsorbents demonstrated a significant adsorption capacity for the Cd (II) ion.

**Figure 13:**
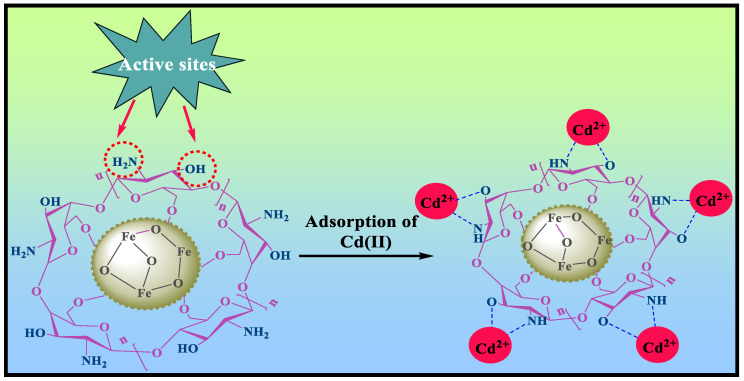
Proposed mechanism for Cd (II) capture by MCS composite [78].

The amines and secondary alcohol functional groups are the major sites for complexing, according to the FTIR analysis of the magnetic chitosan composite before and after Cd (II) adsorption (Figure 13). There is a slight shift in the position of various bands in the MCS after the adsorption of the metal ion, Cd (II). From the XRD data, there was a slight shift in the diffraction peaks after the adsorption of Cd (II). Also, from the magnetization results, the decrease in magnetization indirectly asserted the formation of a composite between CS and MNPs.

The oxygen of the alcohol and the nitrogen of the amino group has a pair of electrons that can add themselves to a proton by coordinated covalent bonds. While nitrogen has a larger capacity to donate its pair of electrons to a cadmium ion to form a complex through a coordinated and covalent bond, oxygen has a stronger attraction of the electron pair by the atom nucleus. This leads us to suggest that the mechanism shown in Figure 13 governs the formation of the complexes between Cd (II) and adsorbent. In this mechanism, the cadmium ion acts as a Lewis acid that may take in electron pairs due to its vacant orbitals. The amine and hydroxyl groups, on the other hand, which have non-shared electron pairs, serve as Lewis bases by giving their electron pair.

## 5. Conclusions

In the present study, magnetic chitosan composition was synthesized via a green route and thoroughly characterized by a variety of physical-chemical techniques. Afterward, the composite materials were successfully utilized for the adsorption of Cd (II). Elaborate studies on the kinetics and thermodynamic aspects of adsorption were carried out.For comparison, MNPs were also used as adsorbents for Cd (II). MCS showed excellent and high adsorption capacity compared to native MNPs. The adsorption capacity values of 426 mg/g for MCS and 290 mg/g for NPs were observed. The BET surface area values of MCS and NPs were found to be 178 and 105 m^2^/g, respectively. Both adsorbents showed second-order kinetics. The optimum process parameters include an adsorbent dose of 0.05 g/L, a contact time of 50 min, and a pH value of 6.0 with an initial Cd (II) concentration of 100 and 50 mg/L. The adsorption processes on both the adsorbents were feasible and spontaneous as evident from the thermodynamic parameters. The composite material showed good regeneration capacity with an 80.44% removal tendency up to the seventh cycle while MNPs showed low removal capacity of 42% by the seventh and the last cycle. This research provides some insights into the aspects that influence the design of adsorbents with superior performance and easy recovery for Cd (II) ion absorption. Magnetic composites have promising applications in water treatment. Its outstanding selective adsorption considerably improves the material’s adsorption efficiency, and it may be specially treated for wastewater enriched with various compounds. Furthermore, the magnetic chitosan adsorption material is easy and simple to recycle, with a high recycling rate. These benefits can lower wastewater treatment costs and increase economic efficiency. So, from the present study, it is concluded that the green synthesized MCS composite with MNPs enhanced the stability of hybrid material and also improved the removal capacity of Cd (II) with good regeneration ability and effective field study results.

## Figures and Tables

**Table 1:** Surface parameters of MNPs and MCS adsorbents before cadmium adsorption.

Parameters	MNPs	MCS
BET specific surface area (m^2^/g)	105	173
Langmuir surface area (m^2^/g)	201	537
Average pore size (Å)	64.36	49.77
Total pore volume (cc/g)	0.3410	0.4305

**Table 2:** The relative adsorption capacity of various adsorbents for Cd (II).

S. No.	Materials	Q_e_(mg/g)	Reference
1	Egg-albumen-formaldehyde-based magnetic polymeric resin	149.3	[56]
2	Mesoporous magnetic nanocomposite	158.68	[57]
3	Amino-decorated magnetic metal-organic framework	693.0	[58]
4	Carboxymethyl chitosan/sodium alginate/graphene oxide@ Fe_3_O_4_ beads	86.28	[59]
6	Poly(γ-glutamic acid) modified magnetic Fe_3_O_4_-GO-(o-MWCNTs) hybrid nanocomposite	625.00	[60]
7	Citric acid- and Fe_3_O_4_-modified sugarcane bagasse	33.2	[61]
8	Fe_3_O_4_@Biuret-formaldehyde pre polymeric resin	92.6	[62]
9	Fe_3_O_4_/FeMoS_4_/MgAl-LDH nanocomposite	140.50	[63]
10	Fe_3_O_4_@PDA microspheres	296.4	[64]
11	Fe_3_O_4_/SiO_2_/PP	30.1	[65]
12	Fe_3_O_4_ nanoparticles	290	This work
13	Fe_3_O_4_/Chitosan composite	426	This work

**Table 3:** The relative adsorption performance of MCS-based adsorbents for heavy metal ions removal.

S. No.	Materials	Heavy Metals	Q_e_(mg/g)	Reference
1.	Magnetic chitosan composite	Ni(II) Cu(II) Pb(II)	108.9 216.8 220.9	[35]
2.	Magnetic chitosan nanocomposites modified with graphene oxide and polyethyleneimine	As(V) Hg(II)	220.26 124.84	[66]
3.	Chitosan magnetic beads modified with cysteine glutaraldehyde Schiff’s base	Cu(II) Cr(VI)	156.49 138.53	[67]
4.	PEI-grafted magnetic gelatin	Pb(II) Cd (II)	341 321	[68]
5.	Magnetic chitosan/polyethyleneimine embedded hydrophobic sodium alginate composite	Cr(VI) Cu(II)	87.53 351.03	[69]
6.	Magnetic graphene oxide/chitosan composite beads	Ni(II)	80.48	[70]
7.	Magnetic Fe_3_O_4_/Chitosan nanoparticles	Pb(II) Cd (II)	79.24 36.42	[54]
8.	Magnetic chitosan/graphene oxide (MCGO) materials	Pb(II)	76.94	[71]
9.	Xanthate-modified cross-linked magnetic chitosan/poly(vinyl alcohol) particles	Pb(II) Cu(II)	59.855 139.797	[72]
10.	Magnetic anaerobic granule sludge/chitosan composite	Pb(II) Cu(II)	97.97 83.65	[73]

## Data Availability

Data are available upon request.

## Supplementary Materials

The following supporting information can be downloaded at: https://www.mdpi.com/article/10.3390/polym15214339/s1, Figure S1: Preparation of Fe_3_O_4_ nanoparticles using kaphal leaf extract, Figure S2: Preparation of chitosan/Fe_3_O_4_ composite, Figure S3: (a) Langmuir curve, (b) BET multipoint of MNPs and MCS, Figure S4: EDX images of (a) MNPs and, (b) MCS composite, Figure S5: title (a) IPD, (b) Elovich kinetic curves for MNPs and MCS composite, respectively, Figure S6: (a)Temkin, (b) D-Radsorption isotherm curves for MNPs and MCS composite respectively, Figure S7: (a) Langmuir, (b) Freundlich non-linear isotherm curves for MNPs and MCS composite respectively, Table S1: Kinetic parameters of PFO, PSO, IPD, Elovich models for Fe_3_O_4_ and MCS composite, Table S2: Adsorption parameters of four isotherm models, Table S3: Thermodynamic parameters for MNPs and magnetic chitosan composite under optimum conditions title; Table S4: Effect of coexisting ions on the adsorption of Cd(II) by MCS composite.
