# Supplementary material for: Myrica esculenta Leaf Extract—Assisted Green Synthesis of Porous Magnetic Chitosan Composites for Fast Removal of Cd (II) from Water: Kinetics and Thermodynamics of Adsorption
Source: Polymers (Basel). 2023 Nov 6;15(21):4339. doi: 10.3390/polym15214339 (PMC10649474; doi:10.3390/polym15214339)
Supplement: Supplementary file 1 [file polymers-15-04339-s001.zip › polymers-2615475-supplementary.pdf]

polymers

MDPI

Article

Myrica esculenta Leaf Extract—Assisted Green Synthesis of Porous Magnetic Chitosan Composites for Fast Removal of Cd (II) from Water: Kinetics and Thermodynamics of Adsorption

Anjali Yadav [1], Sapna Raghav [2], Nirmala Kumari Jangid [1,*], Anamika Srivastava [1], Sapana Jadoun [3], Manish Srivastava [4,*] and Jaya Dwivedi [1]

[1] Department of Chemistry, Banasthali Vidyapith, Banasthali 304022, India
[2] Department of Chemistry, Nirankari Baba Gurubachan Singh Memorial College, Sohna 122103, India
[3] Departamento de Química, Facultad de Ciencias, Universidad de Tarapacá, Avda. General, Velásquez, Arica 1775, Chile
[4] Department of Chemistry, University of Allahabad, Prayagraj 211002, India
* Correspondence: nirmalajangid.111@gmail.com (N.K.J.); sagermanish1@gmail.com (M.S.)

Synthesis of Fe_3O_4 Nanoparticles

$FeCl_3{\cdot}6H_2O$ (0.08 M) and $FeCl_2{\cdot}4H_2O$ (0.04 M) were mixed in 100 mL water, and the temperature was gradually raised to 80 °C under refluxing condition in a N_2 atmosphere with continual mechanical stirring at 2000 rpm.Additionally, 5 mL of leaf extract was added and the temperature was maintained at 80 °C for 15 minutes. Instantaneously, 30 mL of a 25% NaOHsolution was added to the reaction mixture, which was then maintained at 70 °C for an additional 30 minutes. After stirring for 30 minutes the reaction was stopped, and the precipitate was collected and washed with deionized (DI) water. The product was oven-dried after being magnetically separated (**Fig. S1**).

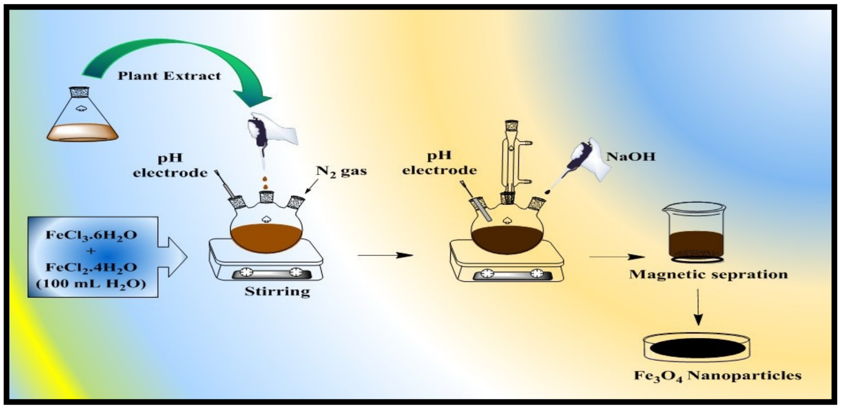

Figure S1. Preparation of Fe_3O_4 nanoparticles using kaphal leaf extract.

Preparation of Fe_3O_4/ChitosanComposite

30 mL of a 2% CH_3COOH solution was added to 1.2 g of chitosan (CS). After that, 4.0 g of freshly synthesized MNPs were added. MNPs weremixedvigorously with CS solution, and the solution was maintained at 80 °C for 30 minutes.To adjust the pH between 8.0 and 10.0,10 mL of NaOH (25 vol%) was added and the stirring was continued for 2 h. The end product was vacuum dried in a vacuum oven at 120 °C for 24 h after being

washed repeatedly with DI water.Thedried product was mashed using mortar and pestle resulting in the end product, namely, Fe_3O_4/chitosan composite (**Fig. S2**).

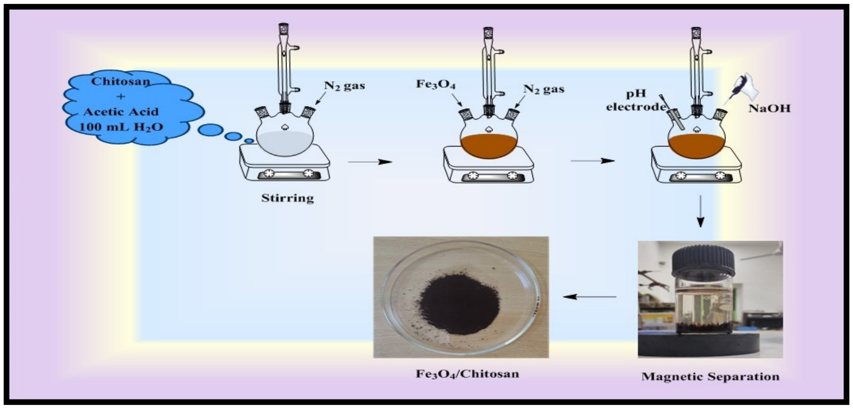

Figure S2. Preparation of chitosan/Fe_3O_4 composite.

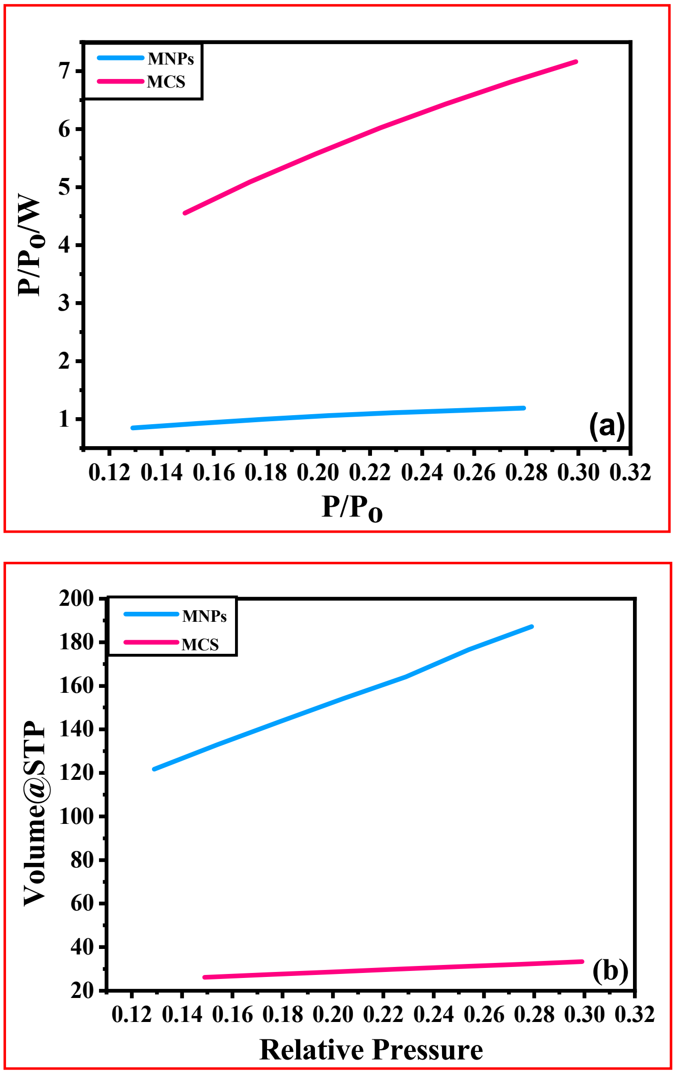

Figure S3. (a) Langmuir curve, **(b)**BET multipoint of MNPs and MCS.

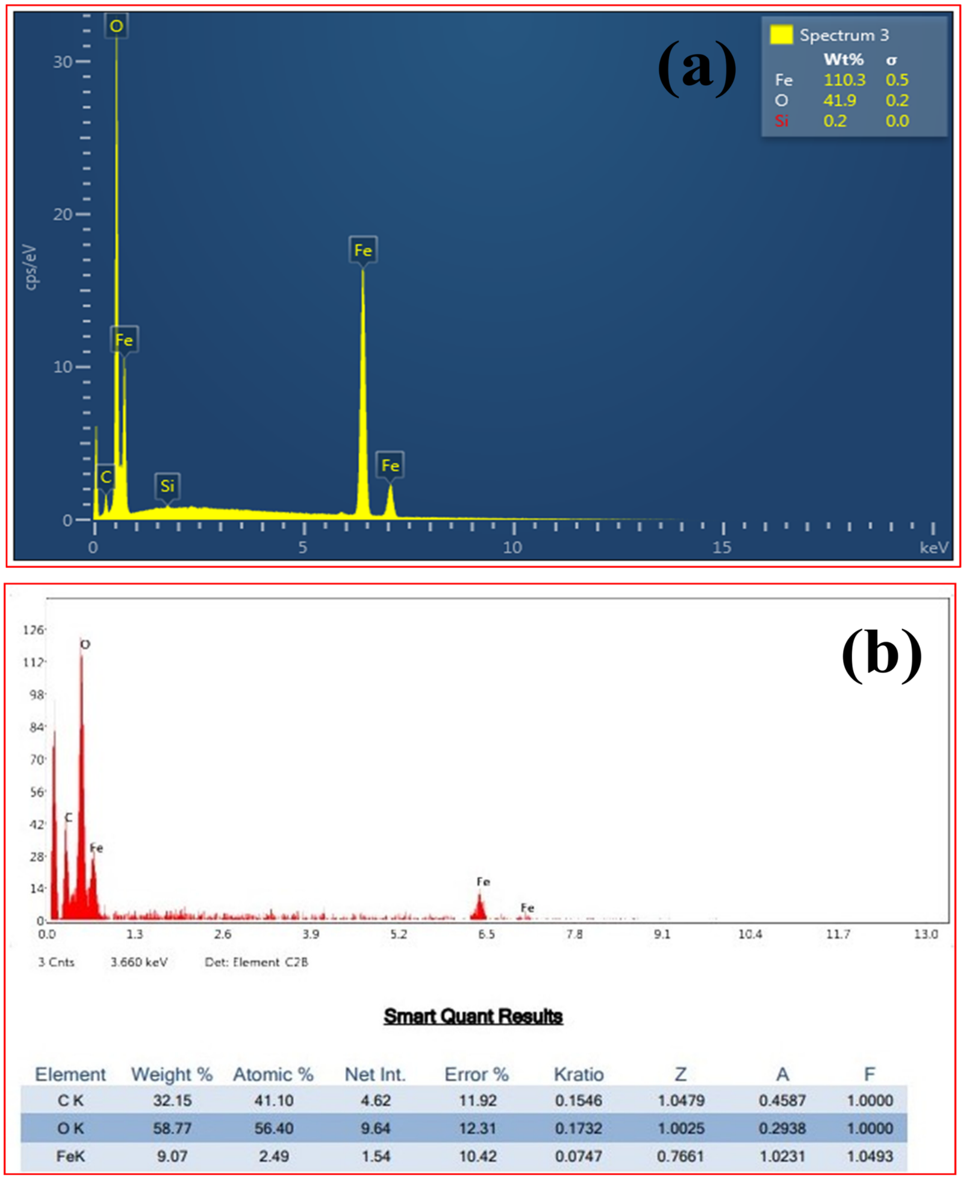

Element	Weight %	Atomic %	Net Int.	Error %	Kratio	Z	A	F
C K	32.15	41.10	4.62	11.92	0.1546	1.0479	0.4587	1.0000
O K	58.77	56.40	9.64	12.31	0.1732	1.0025	0.2938	1.0000
FeK	9.07	2.49	1.54	10.42	0.0747	0.7661	1.0231	1.0493

Figure S4. EDAXimages of (a) MNPs and, (b) MCS composite.

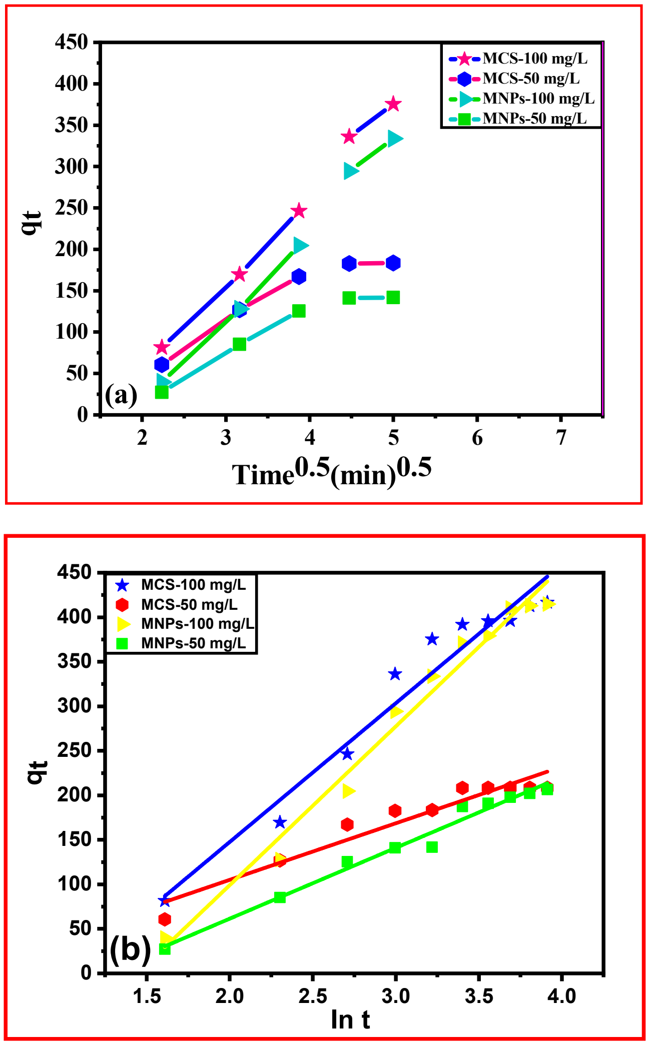

Figure S5. (a) IPD, **(b)**Elovich kinetic curves for MNPs and MCS composite, respectively.

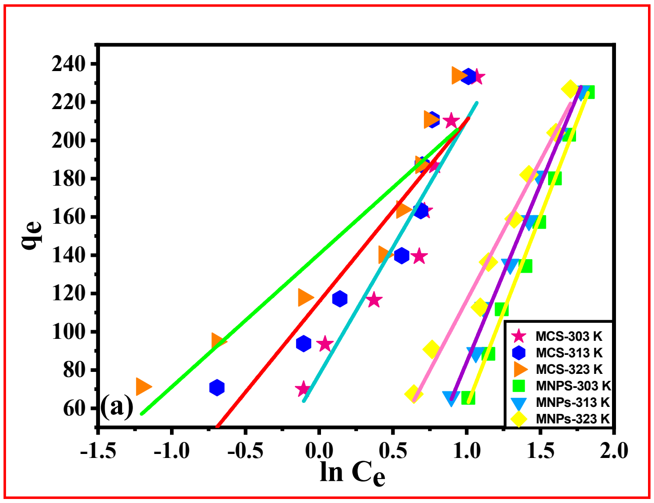

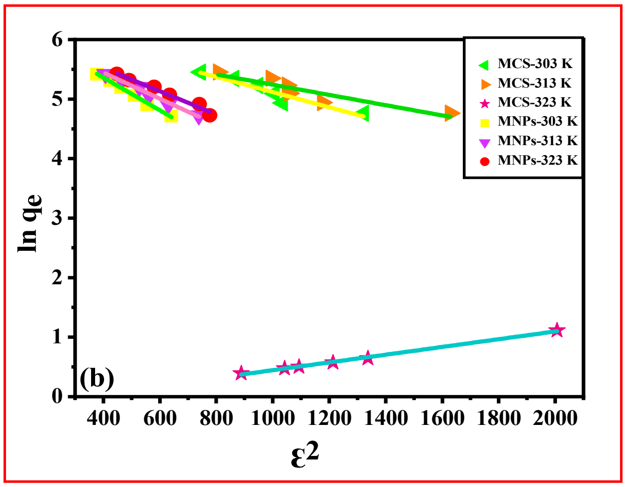

Figure S6. (a) Temkin, **(b)**D-Radsorption isotherm curves for MNPs and MCS compositerespectively.

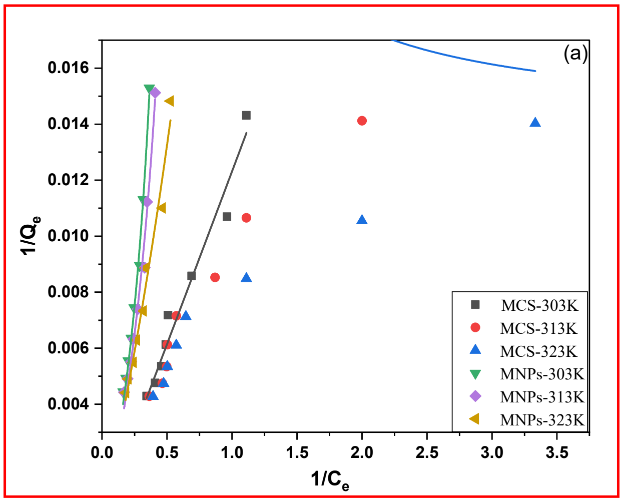

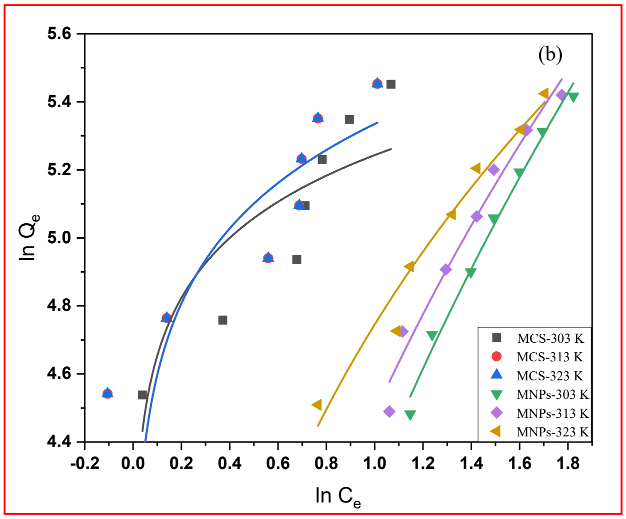

The calculated R^2 values for non-linear is lower as compared to linear. So, our isotherm fitted well with the lineraised equations therefore we did not consider the non-linearised equations.

Figure S7. (a) Langmuir, **(b)** Freundlich non-linear isotherm curves for MNPs and MCS composite respectively.

Table S1. Kinetic parameters of PFO, PSO, IPD, Elovich models for Fe_3O_4 and MCS composite.

**Kinetic**	**Cd(II) concentration (mg/L)**			
	**MNPs**			**MCS**
	**50.0 mg/L**	**100.0 mg/L**	**50.0 mg/L**	**100.0 mg/L**
**PFO**				
**K_1(min^{-1})**	-0.00183	-0.00255	-0.00623	-0.00204
**q_e (mg/g)**	343.7759	1268.005	2783.098	639.1441
**R^2**	0.95	0.89	0.86	0.94
**PSO**				
**K_2(g/mg min^{-1})**	0.00012	0.0000959	0.000815	0.00027
**q_e cal(mg/g)**	321.543	578.034	233.644	480.769
**R^2**	0.90	0.98	0.98	0.99
**IPD**				
**K_i (mg/g min$^{-1/2}$)**	43.032	109.969	45.693	109.969
**C (mg/g)**	57.17	212.15	27.15	170.48
**R^2**	0.89	0.99	0.88	0.99
**Elovich model**				
**B**	0.0125	0.00560	0.0157	0.0064
**α**	0.466	0.841	0.898	1.087
**R^2**	0.97	0.97	0.91	0.95

Table S2. Adsorption parameters of four isotherm models.

**Adsorption constants**				**Temperature (K)**		
	**MNPs**			**MCS**		
	**303 K**	**313 K**	**323 K**	**303 K**	**313 K**	**323 K**
**Langmuir**						
**q_m (mg/g)**	207	255	290	426	353	247
**R_L**	14.8	10.1	1.63	0.89	0.175	0.07
**R^2**	0.94	0.93	0.96	0.96	0.93	0.93
**Freundlich**						
**K_F (mg/g)(L/mg)$^{1/n}$**	19.80372	26.28741	41.32427	85.34057	100.9777	100.9777
**N**	0.733	0.800	0.993	1.075	1.222	1.222
**R^2**	0.97904	0.95542	0.97375	0.94064	0.92481	0.92481
**Temkin**						
**K_T (L/mg)**	0.499	0.5782	0.815	1.794	3.396	7.580
**β (J/mol)**	199.28	185.86	146.10	132.85	94.68	69.43
**R^2**	0.99	0.99	0.974	0.92	0.85	0.85
**D-R plot**						
**q_m (mg/g)**	640.69	557.18	546.16	598.75	447.12	418.13
**β (mol^2/kJ)**	0.00275	0.00219	0.00195	0.00128	0.00119	0.000651
**E (kJ/mol)**	19.06925	21.36869	22.64554	27.95085	27.95085	-
**R^2**	0.99334	0.98579	0.96761	0.90068	0.81441	0.99604

Table S3. Thermodynamic parameters for MNPs and magnetic chitosan composite under optimum conditions.

Adsorbent	Temperature (K)	Thermodynamic parameters		
		ΔH^0 (kJ/mol)	ΔS^0 (kJ/mol·K)	ΔG^0 (kJ/mol)
MNPs	**283**	449.63	1.642	-15.29
	**293**	671.74	2.386	-27.57
	**303**	645.92	2.341	-63.63
MCS	**283**	528.28	1.996	-36.59
	**293**	528.28	2.037	-68.73
	**303**	611.35	2.333	-95.74

Effect of coexisting ions

In the actual situation, contaminated water contains a variety of ions. They would compete for adsorption sites when they are in a coexistence system. Hence, it is necessary to carry out competitive adsorption tests to investigate the influence of target metal ion onto the prepared adsorbents competing with other ions. It is well-known that these cations and anions always coexist in wastewaters with high concentrations. In this study, an interference examination was done to check that the sorption capacity of Cd(II) is not affected in the presence of interfering ions. Batch sorption studies with common ions (NO^{-}_{3} , Cl^-, SO_4^{2-}, Na^+,K^+) were carried out to examine the sorption selectivity of prepared adsorbent (Table S4). Table S4 clearly shows that these ions have no or minimal influence on Cd(II) sorption, indicating that the adsorbent has a high degree of sorption selectivity.

Table S4. Effect of coexisting ions on the adsorption of Cd(II) by MCS composite.

Coexisting ions	Amount of coexisting ions (mg/L)	% Removal	
		MNPs	MCS
Nitrate	100	94.04%	97.32%
Chloride	100	93.87%	97.2%
Sulphate	50	94.5%	97.45%
Sodium	50	93.2%	96.7%
Potassium	50	93.5%	96.8%
